# Association between Moderate Alcohol Consumption and Subjective Quality of Life in Spanish Young Adults

**DOI:** 10.3390/nu15030750

**Published:** 2023-02-01

**Authors:** Iván Vicente-Castro, Noemí Redondo-Useros, Ligia E. Díaz-Prieto, Sonia Gómez-Martínez, Ascensión Marcos, Esther Nova

**Affiliations:** Immunonutrition Research Group, Department of Metabolism and Nutrition, Institute of Food Science and Technology and Nutrition (ICTAN)—CSIC, 28040 Madrid, Spain

**Keywords:** alcohol consumption, young adults, quality of life

## Abstract

Background: For the last 25 years, the debate on the benefit–risk balance of moderate alcohol consumption has been ongoing. This study explored the relationships between the pattern of alcohol consumption and subjective quality of life in healthy adults. Material and Methods: Participants were 247 healthy adults aged 25–45 years, with a moderate alcohol consumption, classified in three groups of alcohol intake: None (N = 37; <0.7 g/day), Low (N = 87; 0.7–<5 g/day) and Medium (N = 123; 5–16 g/day in women and 5–28 g/day in men). Information was collected with questionnaires including: alcoholic beverage frequency and pattern, quality of life (SF-36v2), anxiety, depression, health condition, adherence to the Mediterranean diet, physical activity and sleep time. Results: Mean (SD) age of participants was 35.9 (6.3). In the Medium group, the mean alcohol intake was 10.98 ± 4.84 g/day on weekly bases and 24.7 ± 15.2 g/day on weekend days. Beer was the main contributor to total daily alcohol consumption. The percentage of subjects in the Medium group that showed a weekend average intake higher than moderate was 58.5% in exclusively weekend consumers and 48.2% in those who are not exclusive-weekend consumers (*p* = 0.278). Regarding markers of quality of life and mental health, the results did not show any significant association with alcohol consumption. In conclusion, in subjects that show weekly bases moderate intake of alcohol, weekend-day consumption levels can be high; however, no association of an overall moderate alcohol consumption pattern with quality of life was found.

## 1. Introduction

The benefit–risk balance of moderate alcohol consumption has been widely debated over the last 25 years. On the one hand, it has been associated with positive effects on health, specifically on the cardiovascular and immune systems [1], and it is a habit ingrained in most countries’ cultures, playing an important role in socialization and, therefore, in health-related quality of life (HRQoL). On the other hand, the benefit–risk balance of alcohol consumption depends not only on the amount consumed, but also on several factors, such as the type of alcoholic beverages (fermented versus distilled), the frequency of consumption, the context in which consumption occurs (in social groups or solitude, accompanied by food or not and what type of food), the quantity in relation to the time used for its consumption (binge drinking), and the individual tolerance, which may depend on the efficiency of ethanol metabolism by the enzyme alcohol dehydrogenase or by the particular health condition of each individual [2,3]. Focusing on generally healthy adults and excluding pregnant and lactating women, the most extended conception of a moderate consumption of alcohol has been established at ≤2 drinks/day for men and 1≤ drink/day for women [4,5]. However, when examining published studies on the relationship between alcohol consumption and health, the criteria used for subjects’ inclusion vary among studies. A common practice, until recently, was to include all amounts of alcohol below the World Health Organization (WHO) threshold for heavy drinking, set at 40 g/day for men and 24 g/day for women [6].

Regarding the use of alcoholic drinks in a population, volume is often estimated with sales data, but this does not provide individual-level alcohol consumption and pattern of consumption; hence, it is not useful to link pattern of consumption with alcohol-related harm [7]. On the other hand, population survey methods have several issues that affect the reliability and validity of the data, such as the amplitude of the recall period, the type of questions, or the mode of interview (i.e., face to face versus by telephone) [8]. Using a questionnaire based on beverage specific quantity and frequency questions has been identified as the most appropriate instrument for measuring average volume of alcohol consumption [7]. Few studies have been published in recent years on Spanish adults using this type of instrument. A population-based study of the Spanish pattern of alcohol consumption was carried out using the Health National Survey 2011–2012 [9], and another one with alcohol consumption was collected as part of a diet history at years 2008–2010 and at follow up, three years later [10]. Both studies showed that subjects and populations change their drinking pattern over the years to accommodate to the socioeconomic and health status of a particular period. However, both of them used the cut-off points of 40 g/day for men and 24 g/day for women to separate moderate and heavy drinking, which is higher than the moderate amount most agreed on by experts on alcohol research (5). In a previous study conducted by a research team in Spanish subjects aged 55–85 years, moderate alcohol consumption, as defined in the mentioned terms, was associated with a better score in the vitality and mental health dimensions of the SF-36 questionnaire used in the assessment of HRQoL [11]. Since alcohol abuse is known to be related with poorer HRQoL [12,13], it seems worth exploring the relationship of HRQoL with different alcohol consumption ranges interpreted as moderate. Information on HRQoL in Spanish subjects with an alcohol habit within the range of one and two standard drinks for women and men, respectively, has not been found in the literature.

Therefore, the aim of this study is to advance our understanding of the relationships between alcohol consumption patterns (quantity, type of drink and occasion/context) and subjective quality of life in a sample of Spanish adults, generally considered as healthy, with a moderate consumption habit.

## 2. Materials and Methods

### 2.1. Experimental Design

This is an observational and cross-sectional study based on the ALMICROBHOL study population [14], which was based on an incidental sample, not necessarily representative of the general population. The ALMICROBHOL study was aimed to analyze the associations between alcohol consumption and the intestinal microbiota in healthy adults taking into consideration potential confusion factors, including health-related issues and lifestyle factors.

### 2.2. Study Subjects

A total of 272 adults aged between 25 and 45 years with a body mass index (BMI) between 18.5 and 35 kg/m^2^ were recruited through advertisements in the university area and in several enterprises that disseminated the study among their employees as a health-promotion campaign. Mean (±SD) age of study participants was 35.9 ± 6.3. Exclusion criteria were: (1) pathological conditions such as type 1 diabetes mellitus, cancer; liver, heart, kidney or lung disease, brain or mental disorders, congenital metabolic diseases, autoimmune diseases, inflammatory bowel diseases, previously diagnosed human immunodeficiency virus infection (HIV), Cushing’s syndrome or diagnosed food intolerances; (2) use of chronic medication; (3) antibiotic use in the last two months; (4) to be on a prescribed weight reduction diet or an exclusion diet for food intolerance; (5) having undergone a surgical procedure in the last month.

From the total 272 recruited volunteers, 11 subjects dropped out from the study due to personal issues and did not complete the two study visits, resulting in a final sample size of 261 subjects (134 males and 127 females). Informed consent was obtained from each subject prior to participation, and ethical approval was obtained from the Ethics Committee of Puerta de Hierro University Hospital (Madrid, Spain), and the Bioethics Committee of the Spanish National Research Council (CSIC). The study was carried out according to the Declaration of Helsinki (64th General Assembly, Fortaleza, Brazil, October 2013) and the Spanish law 14/2007 on Biomedical Research.

### 2.3. Study Visits and Questionnaires

During the study, subjects came to the research center (ICTAN) on two occasions to participate in individual face-to-face interviews with trained nutritionists who collected data on their lifestyle habits. In the first visit, questionnaires were completed to collect information on demographic characteristics and socioeconomic status (SES), overall health status, diagnosed diseases, symptoms, non-chronic drug administration, smoking habit, sleep habit, quality of life and alcohol consumption. Anthropometric parameters (height, body weight and waist circumference) were also measured, and body composition analysis with use of bioimpedance method was performed. Questionnaires for mental health and bowel habits were completed at home and handed in at the second visit, when physical activity and dietary habits information was collected through the respective questionnaires in a new interview with the researcher. Each visit took approximately one hour.

### 2.4. Alcohol Intake Assessment

Frequency of drinking was assessed using an ad hoc frequency recall questionnaire on alcoholic beverage consumption based on the modified SUN survey questionnaire [15,16]. The questionnaire recorded the intake of wine, beer, cava, cider, liquors, spirits, both pure and as combination with refreshments, by estimations made by the respondent over the last year with tips and prompts from the interviewer to maximize the quality of the data collected. The most common dose sizes for each of the beverages were considered. Questions on drinking behavior (drinking during or outside meals, with friends and family or alone, etc.) were also included. The frequency of intake of each drink was registered using a continuous scale as follows: never or almost never (0 to once every 2 months), 1 to 3 times per month, 1 to 3 times per week or number of times per day. Habitual intake was recorded for weekdays and separately for weekends. Total alcohol intake (g/day) was calculated using average grams of alcohol content per 100 mL of each alcoholic beverage (wine: 11 g, beer: 4.5 g, cider: 5 g, liquors and spirits: 40 g). Thus, the following alcohol consumption groups were established: (1) “None”: <0.7 g/day of alcohol; (2) “Low”: 0.7–<5 g/day of alcohol; and (3) “Medium”: 5–16 g/day of alcohol in women and 5–28 g/day of alcohol in men. The “Regular drinkers” group consists of individuals in the “Low” and “Medium” groups. These cut-off points refer to less than one drink in 15 days (“None”), between one drink every 15 days and one drink every other day (Low) and up to one can of beer (330 mL) or two cans of beer for women and men, respectively, per day (“Medium”). According to these alcohol criteria, 247 subjects (130 males and 117 females) were finally selected to participate in this study.

### 2.5. Health and Quality of Life Assessment

General health status, diagnosed diseases, symptoms, drug treatments, smoking habits and sleep quality and time were assessed using the corresponding items of the Spanish National Health Survey [17]. Chronic disease prevalence for each individual was calculated considering symptoms and medication related with the following: hypertension, varicose veins, arthrosis, arthritis, rheumatism, chronic back pain, chronic allergy, asthma, chronic bronchitis, diabetes mellitus type 2, stomach or intestinal ulcers, urinary incontinence, high cholesterol, cataracts, chronic dermatological problems, chronic constipation, depression or mental illness, migraines, hemorrhoids, osteoporosis and anemia. Subjects were classified as “no symptoms”, “symptoms for any disease” or “symptoms and medication for any disease”.

Quality of life was assessed using the self-report Short Form-36 Health Survey questionnaire version 2 (SF-36v2) [18]. This is a subjective health perception test that measures the specific dimensions of physical function, physical role, physical pain, general health, vitality, social function, emotional role and mental health. The items corresponding to each scale were summed and the transformed scores (scale 0–100) were calculated using the following formula: Transformed scale = ((raw score − lowest possible score)/possible range) × 100. The State-Trait Anxiety Inventory (STAI) was used to assess the emotional state [19]. The Center of Epidemiological Studies-Depression Scale (CES-D) was used to assess the level of depression [20]. The STAI in the state subscale consists of a total of 20 items with a 4-point Likert response system according to intensity (1 = almost never/not at all; 2 = somewhat/sometimes; 3 = quite often; 4 = very much/almost always). The total score ranges from 20 to 80 points, where a higher score indicates higher levels of anxiety. The CES-D consists of 20 items assessing the presence of symptomatic depressive manifestations in the past week. The score range, after recoding the reverse-coded items, is between 0 and 60, where a higher score indicates higher severity of depressive symptoms.

For the SES classification, interviewer-assisted self-estimation of total capital owned was used as follows: (1) “low-intermediate”: income between 10,000 and 50,000 EUR/year; (2) “intermediate-high”: income between 50,000 and 200,000 EUR/year; and (3) “high”: income above 200,000 EUR/year.

### 2.6. Lifestyle Assessment (Physical Activity and Dietary Habits)

Physical activity was assessed using the Minnesota Leisure–Time Physical Activity Questionnaire (MLTPAQ, Spanish version). For each individual, METs (metabolic standard units) were estimated using the coefficients published in the Compendium of Physical Activities [21] and were computed for weekly periods. Estimated METs include only regular activities (daily, weekly or monthly practiced), while occasional activities were not considered.

Dietary habits of each subject were assessed using the 14-item Mediterranean Diet Adherence Screener (MEDAS) questionnaire [22], excluding the question referring to wine consumption, since it was involved in the creation of the dependent variable “alcohol consumption groups”.

### 2.7. Measurement of Anthropometric Values and Bioimpedance Analysis

Height (Soehnle stadiometer, Hamburg, Germany, accurate to 0.5 cm), waist circumference (Seca 201 tape, Hamburg, Germany, accurate to 0.1 cm), and body weight (InnerScan BC 601 scale, Tanita Europe B. V., Amsterdam, The Netherlands, accurate to 0.1 kg) were measured in each individual without shoes and with light clothing. For bioimpedance analysis, an InnerScan BC 601 device (Tanita Europe B. V., Amsterdam, The Netherlands) with 8 electrodes was used. BMI was calculated using the formula: weight (kg)/height (m)^2^. Since BMI does not represent an accurate measurement of body fat, the optimal body fat percentages were considered separately for men and women, and the subjects were divided into two body fat status groups (BFG): (1) normal body fat percentage (21–32% for women; 10–20% for men); and (2) high body fat percentage (>32% for women; >20% for men). Cut-off criteria for body fat percentages were taken from the Tanita guidelines, which in turn are taken from Gallagher et al. [23].

### 2.8. Statistical Analysis

The Kolmogorov–Smirnov normality test was used to check the normality of the variables in the groups defined by male and female sex, as well as in the alcohol consumption groups (None, Low and Medium). Logarithmic transformation was applied for data normalization (physical activity and hours of sleep). To characterize the study population, descriptive statistics were calculated for the demographic, anthropometric and lifestyle variables of the study subjects divided by sex and stratified according to alcohol consumption groups, using parametric or non-parametric statistics depending on the sample size. Student’s *t* test and ANOVA were used for normally distributed variables, Mann–Whitney U test and Kruskall–Wallis for non-normally distributed variables and Chi-square test for categorical variables. The effect of alcohol consumption on quality of life (depression, anxiety and SF-36 dimensions) and lifestyle variables (physical activity and sleep hours) was analyzed using a univariate general linear model with the alcohol consumption group, sex, BFG and SES as fixed variables. Similarly, the relationships between alcohol intake from the different types of beverages and quality of life and lifestyle variables were analyzed using a general linear model adjusted for sex, BFG and SES.

The sample size of the ALMICROBHOL study was calculated for a main outcome related with intestinal microbiota composition and has proven too small to provide an adequate statistical power for the QoL variables presented in the current work. Regarding calculating an a priori required sample size for an α-error = 0.05, a power = 0.70 and using the mean and SD values for the variable, physical function, obtained in the current work, the smallest number of subjects to probe a significant difference between three groups (F test) with one-way ANOVA of fixed effects is 687 total subjects.

Data analysis was performed using SPSS v.25 Software (IBM Corp., Armonk, NY, USA), considering a value of *p* < 0.05 as statistically significant.

## 3. Results

Demographic, anthropometric and lifestyle characteristics of study subjects are shown in Table 1. No significant differences between the alcohol consumption groups were found in men. Among females, significant differences were only observed in age, with abstainers being on average 4 to 5 years older than the two regular consumer groups (*p* = 0.010).

The daily consumption of different alcoholic beverages, the average daily alcohol intake and that on weekend days, in Low amount consumers and Medium amount consumers, are shown in Table 2. Only 2 people out of 210 reported consuming any cider (small amounts), and therefore, no results are presented. Beer was the most consumed beverage in all drinking groups (g/day). In the Low consumption group, wine contributed 5.3% more to total daily alcohol consumption compared to the Medium consumption group (19.6% (0.0–55.0) vs. 14.3% (0.0–45.1)), in which beer clearly predominated. Noticeably, consumption of all beverages on the weekends exceeded the average consumption during the week. The same data stratified by sex, also showed that beer was the most consumed beverage in the different consumption groups, both in men and women. Beer consumption in the Medium group in both sexes was very similar (6.47 ± 5.03 g/day in men vs. 6.53 ± 4.34 g/day in women, data not shown), while consumption of distilled beverages was higher in men than in women (1.77 ± 2.55 vs. 0.83 ± 1.17 g/day, *p* = 0.020). Both men and women consumed more distilled beverages in the Medium consumption group than in the Low consumption group.

The occasion and social context in which alcohol is consumed are presented in Table 3. In both consumer groups, the percentage of people who consumed a different amount of alcohol on weekend days and on weekdays was high; however, this was significantly higher in the Medium group compared to the Low group (89.4% vs. 80.4%, *p* ≤ 0.001). Subjects in the Low consumption group mostly reported alcohol consumption restricted to weekend days, differently from the Medium consumption group (65.5% vs. 33.3%, *p* ≤ 0.001), that mainly presented consumption on all weekdays. The frequency of sporadic alcohol abuse also differs in both consumption groups. While 52.3% of the subjects in the Low consumption group never consumed 5 drinks or more in a single session, only 18.0% of the subjects in the Medium consumption group did not engage in this behavior over the last year (*p* ≤ 0.001). Similarly, subjects in the Medium consumption group reported getting drunk more often than the Low consumption group (*p* = 0.012). Specifically, 35% of the subjects in the Medium consumption group reported getting drunk between one and four times per year. In addition, 33.3% of them had declared themselves as exclusive weekend consumers (data not shown). In the sex-stratified analysis of frequency of drunkenness, the difference was significant only for women. While 70.8% of the women in the Low consumption group were never drunk, this was true only for 40.0% of the women in the Medium consumption group (*p* ≤ 0.001).

The average daily consumption of alcohol is higher for those who drink both on the weekdays and weekend days compared to those with the weekend-only habit (Low consumption: 3.02 ± 1.33 vs. 2.16 ± 1.32 total alcohol g/day, *p* = 0.003; Medium consumption: 11.76 ± 4.95 vs. 9.44 ± 4.25 total alcohol g/day, *p* = 0.006). However, when focusing on the consumption made during the weekend (Table 4), total alcohol consumption on each weekend day on the Medium group was higher for those with a weekend-only drinking habit in relation to individuals who drank both on weekend days and weekdays (29.65 ± 12.61 vs. 22.23 ± 15.76, *p* = 0.005), and it seems to be due both to a higher consumption of beer and wine. On an individual basis, the weekend average intake was higher than moderate in 58.5% of subjects of the Medium group that drank alcohol exclusively on the weekends and in 48.2% of subjects who were not exclusive-weekend consumers; the difference between these percentages, however, was not significant (*p* = 0.278). Moreover, no differences were observed in the frequency of drinking five alcoholic beverages or more in a single sitting nor in the frequency of drunkenness between individuals with or without a weekend-only drinking pattern. Finally, in our sample, a higher percentage of women in the Medium group have a higher than moderate consumption on the weekend days (74.5%) compared with this percentage among males (37.7%). The same was true for 20% of the regular consumers studied (Low and Medium group subjects).

The evaluation of the quality of life and mental health measures consumption showed no significant effect of the average amount of alcohol intake (Table 5). Moreover, no relationship between these variables and the quantity consumed of each type of beverage was found (data not shown). The most influential factors explaining the self-assessment of physical and mental health in the study subjects were other factors introduced in the model, mainly sex for mental health variables and excess body fat to explain variability in physical function. Similarly, sex was significantly related to the amount of energy expenditure on physical activity, but there was no influence of the amount of alcohol consumption on physical activity in the studied individuals. Neither was alcohol amount related to sleep hours per day.

## 4. Discussion

This study characterized the pattern of moderate alcohol consumption in 247 supposedly healthy adults and its relationship with subjective quality of life. Total alcohol and differential consumption by beverage type were estimated, and the occasion/context of alcohol consumption was also described according to the subject’s report.

The distribution of alcohol consumption by type of beverage in the study groups shows that beer has a much higher contribution to total daily alcohol consumption than wine and distilled beverages. These data are consistent with the alcoholic beverage consumption preferences of Spanish people, which, after the year 2000, has partially shifted some of the previous wine consumption towards beer [24]. According to data from the MAPA (Ministry of Agriculture, Fisheries and Food), overall domestic consumption in 2018 was 7.89 L/capita of wine and 18.1 L/capita of beer, reflecting a 2.3 times higher consumption of beer compared to wine [25]. Moderate alcohol intake from wine during meals is no longer a common feature of the Mediterranean diet in early 21st-century Spain [26].

Regarding the occasion on which alcohol is consumed, the Medium consumption was mostly not restricted to the weekend, especially in women, while the Low consumption was usually restricted to the weekend in both sexes. In any case, the vast majority of people in both groups report different alcohol consumption on the weekend than on weekdays, in agreement with the literature that describes higher consumption on festive days [27]. Historically, this moderate drinking during the week and heavy drinking during the weekend is motivated by a spirit of hedonic drunkenness during the weekend [28]. Thus, reasons for consuming alcohol can force decisions about the appropriate days and contexts to consume it [29]. In the current study, subjects who are not exclusive weekend consumers show a higher total alcohol intake (weekly average), but nevertheless, alcohol intake on weekend days is lower than for those who are exclusive weekend drinkers. This can be interpreted as a more regular pattern of daily consumption in the former. In addition, in the Medium group, exclusive weekend consumption was associated with an alcohol intake on each weekend day of approximately 30 g, which is higher than the consumption defined as moderate, particularly for women. It should be noted that this pattern of drinking could be harmful, especially if maintained over long periods. This behavior was observed in 20% of the subjects classified as regular drinkers, and sex and other individual variables should be considered to judge the harmfulness of this drinking habit in each individual.

The binge drinking pattern was approached in terms of having five or more drinks in a single session, which does not exactly match the definition for “binge” because the time frame was not considered. Nevertheless, this pattern was more common in the Medium consumption group than in the Low consumption group. In addition, the Medium consumption group reported a higher frequency of “drunkenness” than the Low consumption group. Other studies have shown that drinking alcohol and engaging in harmful alcohol behavior is frequent in young adults [30]. Specifically, the study conducted in Spain by Soler-Vila et al. in 2008–2010 indicates that 10% of men and 4.2% of women get drunk, with a higher prevalence among young people aged 18–24 years. The average number of monthly binge drinking episodes was 2.3 for males and 2 for females. Among binge drinkers, 61% were aged 18–34 years, more than 80% were regular moderate drinkers, 25% reported frequent drinking, and 22.8% reported heavy drinking [31]. Heavy drinking tends to occur on the weekends, with moderate or low drinking on most other days of the week [32]. This heavy drinking is associated with an increased risk of both acute (e.g., injuries) and long-term (e.g., alcohol-related disorders) consequences [33]. In this sense, reducing this excessive weekend drinking at social events would help to reduce these acute and long-term consequences.

Regarding lifestyle variables, such as adherence to the Mediterranean diet, smoking habits, weekly hours of physical activity in leisure time or daily hours of sleep, our study showed no differences between the alcohol consumption groups, which are also balanced in terms of the SES and the prevalence of common chronic health issues. This finding indicates that drinking alcohol in moderation in the context of a balanced diet is not particularly related to other specific lifestyle habits, such as those mentioned above, in this specific population sample of early and early middle adults. Anthropometrical variables and body composition were also no different according to alcohol consumption. Although there is no influence of the amount of alcohol consumption on the amount of physical activity in this study, the work of Smothers and Bertolucci [34], performed with 41,104 subjects representative of the United States population has shown that physical activity increases with alcohol intake in a dose-dependent manner, from abstinence to moderate, before decreasing with heavy drinking. In addition, several studies have reported a positive association between outdoor physical activity and moderate alcohol consumption, which is considered as evidence of a health-promoting lifestyle [35,36,37]. In line with these results, a previous study by our research group in a population aged between 55 and 80 years also showed more physical activity in moderate drinkers than in abstainers, which could be due to the fact that, in older people, the prevalence and amount of physical activity practice is more variable than in younger people [11].

Regarding the quality of life and mental health markers assessed in our population, the main results of the study showed no association with total alcohol consumption or with the amount consumed of each type of beverage. The scores obtained for the eight dimensions assessed in the SF-36 questionnaire have values of 70 or higher, on a scale from 0 to 100, where 100 would be the score corresponding to a more positive assessment of health. These are the expected results in a presumably healthy population of young to middle-aged individuals. In terms of mental health, the study subjects have low scores on the STAI and CES-D questionnaire, indicating low levels of anxiety and depressive symptoms, respectively. The study carried out by Puddephatt et al. [38] suggested that the association between alcohol and mental health is more complex than a direct relationship. This is because subjects who met the criteria for a mental health problem reported both no alcohol consumption and moderate consumption. However, none of the participants in our study had a diagnosis of a mental disorder. Despite the well-established association between heavy drinking and all types of dementia, the evidence pertaining to moderate alcohol use and risk of dementia and impaired cognition is somehow contradictory, and the level of evidence and the methodological quality of the reviews published thus far has been judged to be only moderate [39].

Some limitations of our study must be considered. Firstly, this is not an epidemiological study; on the contrary, it is based in the incidental sample of the ALMICROBHOL study. Thus, the sample population is small compared to other studies assessing relationships between alcohol consumption and behaviors and quality of life; however, the methodology used to assess alcohol intake (face-to-face interview) is also different to those studies and increases precision of alcohol consumption estimates. Since those estimates were based on researcher-guided self-reports, the probability of underestimation is lower, especially since no heavy drinkers were included [40]; in addition, the types of cognitive cues used by the interviewer to help the subject recall his/her usual habits were previously trained to minimize the introduction of interviewer bias. On the other hand, participants responded voluntarily to the dissemination methods employed. In this sense, the sample could be biased in some way, i.e., to include people with relatively better health and more free time; however, a substantial number of the interviews were conducted at the volunteers’ workplace of two companies that offered the activity to their employees as an educational campaign to promote good lifestyle habits, thus decreasing the likelihood of such bias.

## 5. Conclusions

In conclusion, our results show that, in this young adult population with good general health, the consumption of different amounts of alcohol within the range considered moderate is not associated with parameters of quality of life, mental health or lifestyle. Given the small size of the sample population, this conclusion deserves further study; however, our results suggest that it is unlikely that a big effect was found in future studies performed in young adult healthy populations assessing the association of moderate consumption of alcohol with quality of life. On the other hand, in relation to the pattern of consumption, among the subjects with an intake that could be classified as moderate (5–16 g/day of alcohol in women and 5–28 g/day of alcohol in men), a relevant percentage of subjects consumed above the moderate range on weekend days, especially women. It is not ruled out that this drinking pattern might be associated with changes in quality of life at later stages, specifically if it is a long-term behavior. Thus, this fact should be considered in studies on moderate consumption in order to not alter the outcomes (e.g., health biomarkers). Population-based studies, especially prospective and with well-defined control groups, are needed to help provide more scientific evidence on the relationship between lifestyle patterns related to alcohol consumption and indicators of health and quality of life.

## Figures and Tables

**Table 1:** Demographic, anthropometric and lifestyle characteristics of the different alcohol consumption groups stratified by sex.

Alcohol Consumption Groups						
Men	None (N = 15)	Low (N = 38)	Medium (N = 77)	*p* ^ϒ^	*p* *	*p* ^¥^
**Age (years)**	37.83 (6.42)	38.05 (6.13)	36.32 (5.71)	0.289	-	-
**BMI (kg/m^2^)**	26.48 (3.05)	26.04 (3.79)	25.37 (2.83)	0.341	-	-
**BFG (%)**						
***Normal weight***	46.7	55.3	55.4	-	-	0.757
***Overweight***	53.3	44.7	44.6			
**Body fat (%)**	19.45 (6.27)	20.04 (6.03)	18.46 (5.18)	0.342	-	-
**Waist circumference (cm)**	90.00 (9.69)	89.22 (9.45)	86.26 (7.68)	0.109	-	-
**Visceral fat index**	8.00 (4.00–9.00)	6.00 (4.00–10.00)	6.00 (4.00–8.00)	-	0.464	-
**Total score MEDAS**	7.07 (2.60)	7.05 (1.86)	7.26 (1.90)	0.847	-	-
**SES (%)**						
***Low (<50,000 EUR/year)***	33.3	23.7	35	-	-	0.683
***Medium (50,000–200,000 EUR/year)***	53.3	50.0	44.2			
***High (>200,000 EUR/year)***	13.3	26.3	20.8			
**Smoking habits (%)**						
***Non-smokers***	0.0	15.8	22	-	-	0.292
***Current smokers***	20.0	13.2	13			
***Former smokers***	80.0	71.1	65			
**Physical activity (kcal/week) ^†^**	5547 (3863)	5151 (3371)	5831 (4337)	0.696	-	-
**Sleep (h/d) ^†^**	7.36 (1.08)	7.24 (0.76)	7.33 (0.80)	0.815	-	-
**Women**	**None** **(N = 22)**	**Low** **(N = 49)**	**Medium** **(N = 46)**	***p* ^ϒ^**	*p* *	***p* ^¥^**
**Age (years)**	38.52 (5.70) ^a^	34.14 (6.47) ^b^	33.60 (6.64) ^b^	0.010	-	-
**BMI (kg/m^2^)**	22.18 (2.31)	23.10 (3.36)	22.58 (2.50)	0.419	-	-
**BFG (%)**						
***Normal weight***	77.3	75.5	79.5	-	-	0.514
***Overweight***	22.7	24.5	20.5			
**Body fat (%)**	25.95 (6.88)	27.99 (7.39)	26.56 (5.26)	0.431	-	-
**Waist circumference (cm)**	72.03 (5.85)	75.10 (8.50)	72.72 (6.47)	0.161	-	-
**Visceral fat index**	3.00 (2.00–4.00)	3.00 (2.00–4.00)	2.50 (1.00–4.00)	-	0.439	-
**Total score MEDAS**	7.68 (1.78)	7.31 (2.00)	7.57 (1.93)	0.696	-	-
**SES (%)**						
***Low (<50,000 EUR/year)***	50.0	69.4	58.7	-	-	0.200
***Medium (50,000–200,000 EUR/year)***	36.4	24.5	39.1			
***High (>200,000 EUR/year)***	13.6	6.1	2.2			
**Smoking habits (%)**						
***Non-smokers***	18.2	24.5	19.5	-	-	0.273
***Current smokers***	31.8	26.5	28.3			
***Former smokers***	50.0	49.0	52.2			
**Physical activity (kcal/week) ^†^**	2802 (2285)	3159 (2176)	3145 (2561)	0.821	-	-
**Sleep (h/d) ^†^**	7.86 (0.85)	7.45 (0.70)	7.17 (1.67)	0.087	-	-
**All**	**None** **(N = 37)**	**Low** **(N = 87)**	**Medium** **(N = 123)**	***p* ^ϒ^**	*p* *	***p* ^¥^**
**Chronic disease Prevalence (%)**						
***None***	37.8	31.0	35.8	-	-	0.568
***Symptoms***	21.6	16.1	22.0			
***Symptoms and medication***	40.5	52.9	42.3			

Continuous variables are presented as mean (SD) and Visceral fat index as median (interquartile range). BMI: Body Mass Index, BFG: % body fat groups, MEDAS: Mediterranean Diet Adherence Screener questionnaire, SES: socioeconomic status, h: hours, d: day. Alcohol consumption groups: None (alcohol consumption <0.7 g/day), Low (alcohol consumption between 0.7–<5 g/day) and Medium (alcohol consumption between 5–16 g/day in women and between 5–28 g/day in men). ^ϒ^ ANOVA test. * Kruskall–Wallis test. ^¥^ Chi-square test. ^†^ Variables transformed to logarithmic scale (Ln). Statistical significance was set at *p* < 0.05. ^a,b^ Different letters indicate significant differences (Bonferroni correction).

**Table 2:** Daily consumption of different alcoholic beverages on a weekly basis and on the weekends.

		Weekly Average		Weekend Average	
		Low (N = 87)	Medium (N = 123)	Low (N = 87)	Medium (N = 123)
Beer	g/day %	1.06 ± 1.33 34.9 (0.0–75.0)	6.50 ± 4.77 67.0 (35.3–87.1)	3.77 ± 6.47 29.0 (0.0–100.0)	13.79 ± 12.84 58.4 (0.0–100.0)
Wine	g/day %	0.71 ± 0.98 19.6 (0.0–55.0)	2.76 ± 3.18 14.3 (0.0–45.1)	1.58 ± 3.22 0.0 (0.0–52.4)	6.15 ± 8.91 0.0 (0.0–48.7)
Spirits	g/day %	0.37 ± 0.65 0.0 (0.0–32.6)	1.42 ± 2.18 6.0 (0.0–17.3)	0.85 ± 2.18 0.0 (0.0–12.5)	4.15 ± 7.65 0.0 (0.0–26.0)
Shandy	g/day %	0.32 ± 0.68 0.0 (0.0–7.6)	0.30 ± 0.95 0.0 (0.0–0.0)	0.94 ± 2.36 0.0 (0.0–6.5)	0.58 ± 2.15 0.0 (0.0–0.0)
Total alcohol	g/day	2.46 ± 1.37	10.98 ± 4.84	7.14 ± 6.93	24.70 ± 15.15

Data presented as mean ± SD (g/day) and median (interquartile range) (%). Alcohol consumption groups: Low, 0.7 to < 5 g alcohol/day; Medium, 5 to 16 g alcohol/day in women and 5 to 28 g alcohol/day in men.

**Table 3:** Social pattern of alcohol consumption.

	Alcohol Consumption Groups			
	Low (N = 87)	Medium (N = 123)	*p*	Regular Drinkers (N = 210)
**Social alcohol consumption (%)**				
***Alone***	1.1	0.8	0.487	0.9
***Almost always alone***	0.0	1.6		0.9
***Almost always accompanied***	8.0	13.1		10.9
***Always accompanied***	90.8	84.4		87.2
**Drinking companion (%)**				
***Friends***	23.0	22.7	0.844	22.6
***Relative***	2.3	4.2		3.4
***Others***	0.0	1.7		1.0
***Friends and relatives***	74.7	71.4		73.1
**Different alcohol consumption on the weekends (%)**				
***Yes***	80.5	89.4	<0.001	85.8
***No***	19.5	10.6		14.2
**Alcohol consumption only on the weekends (%)**				
***Yes***	65.5	33.3	<0.001	53.8
***No***	34.5	66.7		46.2
**Frequency of consumption of 5 drinks or more in one sitting (%)**				
***Never***	52.3	18.0	<0.001	31.9
***4 times or less in 1 year***	30.2	26.2		28.6
***2 times per quarter***	14.0	23.0		19.0
***1 time per month***	3.5	17.2		11.4
***2 times per month***	0.0	10.7		6.2
***4 o 5 times per month***	0.0	3.3		1.9
***8 times per month***	0.0	1.6		1.0
***More than 8 times per month***	0.0	0.0		0.0
**Time when 5 or more drinks are consumed at a single sitting (%)**				
***Any day of the week***	58.6	23.6	<0.001	37.7
***Weekend***	41.4	76.4		62.3
**Situation in which 5 or more drinks are consumed in a single sitting (%)**				
***Alone***	0.0	1.0	0.805	0.7
***Almost always alone***	0.0	0.0		0.0
***Almost always accompanied***	0.0	0.0		0.0
***Always accompanied***	100.0	99.0		99.3
**With whom 5 or more drinks are consumed at a single sitting (%)**				
***With friends***	41.5	49.0	0.533	46.5
***With relative***	58.5	47.0		50.7
***With friends and relatives***	0.0	4.0		2.8
**Frequency of drunkenness (%)**				
***Never***	76.2	55.7	0.012	64.4
***4 times or less in 1 year***	20.2	35.2		28.8
***2 times per quarter***	3.6	4.9		4.3
***1 time per month***	0.0	2.5		1.4
***2 times per month***	0.0	0.8		0.5
***4 or 5 times per month***	0.0	0.0		0.0
***8 times per month***	0.0	0.8		0.5
***More than 8 times per month***	0.0	0.0		0.0

Alcohol consumption groups: Low (alcohol consumption between 0.7–<5 g/day) and Medium (alcohol consumption between 5–16 g/day in women and between 5–28 g/day in men). Regular drinkers: alcohol consumption >0.7 g/day. Chi-square test.

**Table 4:** Weekend consumption of each type of alcoholic beverage and alcohol consumption habits according to exclusive or non-exclusive weekend consumption for both alcohol consumption groups.

	Exclusive Weekend Consumption		
Low consumption	Yes (N = 57)	No (N = 30)	*p*
**Beer (g/day)**	4.20 ± 6.65	2.95 ± 6.13	0.197
**Wine (g/day)**	1.83 ± 3.15	1.10 ± 3.36	0.158
**Spirits (g/day)**	1.12 ± 2.50	0.33 ± 1.27	0.055
**Shandy (g/day)**	0.84 ± 2.10	1.12 ± 2.82	0.301
**Total alcohol (g/day)**	8.00 ± 6.76	5.51 ± 7.08	0.056
**Five drinks or more in a single sitting (%)**			
***Never***	57.9	40.0	0.081
***4 times or less in 1 year***	31.6	30.0	
***2 times per quarter***	7.0	26.7	
***1 time per month***	3.5	3.3	
**Frequency of drunkenness (%)**			
***Never***	82.1	65.5	0.185
***4 times or less in 1 year***	14.3	31.0	
***2 times per quarter***	3.6	3.4	
**Medium consumption**	**Yes (N = 41)**	**No (N = 82)**	***p***
**Beer (g/day)**	16.35 ± 11.33	12.50 ± 13.41	0.059
**Wine (g/day)**	7.86 ± 9.49	5.30 ± 8.53	0.067
**Spirits (g/day)**	5.30 ± 7.26	3.57 ± 7.82	0.118
**Shandy (g/day)**	0.14 ± 0.63	0.80 ± 2.57	0.055
**Total alcohol (g/day)**	29.65 ± 12.61	22.23 ± 15.76	0.005
**Five drinks or more in a single sitting (%)**			
***Never***	26.8	13.4	0.115
***4 times or less in 1 year***	26.8	26.8	
***2 times per quarter***	22.0	23.2	
***1 time per month***	12.2	19.5	
***2 times per month***	7.3	12.2	
***4 or 5 times per month***	0.0	4.9	
***8 times per month***	4.9	0.0	
**Frequency of drunkenness (%)**			
***Never***	70.7	48.8	0.097
***4 times or less in 1 year***	22.0	41.5	
***2 times per quarter***	4.9	4.9	
***1 time per month***	0.0	3.7	
***2 times per month***	0.0	1.2	
***8 times per month***	2.4	0.0	

The g/day value presented is the mean ± SD for any weekend day. Alcohol consumption groups: Low (alcohol consumption between 0.7–<5 g/day) and Medium (alcohol consumption between 5–16 g/day in women and between 5–28 g/day in men). Student’s *t* test for continuous variables and Chi-square test for categorical variables. Statistical significance was set at *p* < 0.05.

**Table 5:** Scoring of mental health and quality of life measures according to alcohol consumption groups.

	Alcohol Consumption Groups			Corrected Model			Alcohol Consumption Group			Significant Factor and its Interaction with Alcohol Group		
	None (N = 37)	Low (N = 87)	Medium (N = 123)	F	*p*	Power (1-β)	F	*p*	Power (1-β)	F	*p*	Power (1-β)
**STAI test (anxiety)**	35.4 ± 10.7	34.0 ± 7.8	33.5 ± 9.0	1.5	0.055	0.980	0.2	0.787	0.087	F_sex_ = 5.9 F_sex*gp_alc_ = 0.5	0.016 0.625	0.679 0.127
**CES test (depression)**	9.2 ± 9.0	6.7 ± 5.2	7.3 ± 6.5	1.2	0.184	0.950	0.7	0.517	0.160			
**SF-36**												
***Physical function***	95.8 ± 6.8	97.3 ± 4.4	97.5 ± 5.8	1.5	0.037	0.987	1.4	0.238	0.307	F_BFG_ = 6.2 F_BFG*gp_alc_ = 0.3	0.013 0.723	0.699 0.101
***Physical role***	94.6 ± 11.5	96.7 ± 10.6	94.2 ± 12.9	0.9	0.557	0.848	0.9	0.402	0.207			
***Physical pain***	88.6 ± 14.0	85.4 ± 13.8	85.2 ± 14.6	1.0	0.475	0.874	2.5	0.083	0.501			
***General health***	82.0 ± 13.5	82.0 ± 13.5	83.6 ± 11.6	0.9	0.684	0.800	0.1	0.943	0.059			
***Vitality***	68.9 ± 16.0	70.4 ± 12.3	70.0 ± 12.2	0.9	0.588	0.837	0.8	0.455	0.184			
***Social function***	89.4 ± 15.5	90.2 ± 14.9	92.7 ± 10.6	1.6	0.022	0.991	0.3	0.716	0.103	F_sex_ = 4.4 F_sex*gp_alc_ = 2.4	0.038 0.096	0.549 0.476
***Emotional role***	93.3 ± 12.9	94.2 ± 12.7	96.2 ± 10.9	1.0	0.426	0.888	0.2	0.784	0.088	F_sex_ = 4.4 F_sex*gp_alc_ = 1.5	0.037 0.233	0.553 0.311
***Mental health***	77.6 ± 13.4	78.4 ± 12.1	79.9 ± 12.1	0.8	0.766	0.762	0.2	0.815	0.082			
**Physical activity (kcal/week) ^†^**	3915.0 ± 3273.3	4029.1 ± 2918.7	4818.4 ± 3976.2	1.7	0.016	0.993	0.2	0.814	0.082	F_sex_ = 15.5 F_sex*gp_alc_ = 0.4	<0.001 0.670	0.975 0.114
**Sleep (h/d) ^†^**	7.7 ± 1.0	7.4 ± 0.7	7.3 ± 1.2	0.8	0.726	0.785	2.8	0.065	0.541			

STAI: State–Trait Anxiety Inventory, CES: Center of Epidemiological Studies—Depression Scale, SF-36: Short Form—36 Health Survey questionnaire, BFG: % Body Fat Groups, gp_alc: alcohol consumption group, h: hours, d: day. Mean ± SD. Alcohol consumption groups: None (alcohol consumption <0.7 g/day), Low (alcohol consumption between 0.7–<5 g/day) and Medium (alcohol consumption between 5–16 g/day in women and between 5–28 g/day in men). ^†^ Variables transformed to logarithmic scale (Ln). Univariate general linear model with alcohol consumption group, sex, BFG and socioeconomic status as factors and mental health and quality of life measures as independent variables. Statistical significance was set at *p* < 0.05.

## Data Availability

The data presented in this study are available on request from the corresponding author, due to privacy restriction.
